# Supplementary material for: Comparative multiomics analysis of cell physiological state after culture in a basket bioreactor
Source: Sci Rep. 2022 Nov 23;12:20161. doi: 10.1038/s41598-022-24687-4 (PMC9686226; doi:10.1038/s41598-022-24687-4)
Supplement: Supplementary file 1 — Supplementary Information 1. [file 41598_2022_24687_MOESM1_ESM.zip › raw data/Transcriptomic raw data/6.Enrichment/1.GO/TE1vsTC1/GSEA/TE1/PROTEIN_HOMOOLIGOMERIZATION/PROTEIN_HOMOOLIGOMERIZATION(GO_0051260).html]

Details for gene set PROTEIN\_HOMOOLIGOMERIZATION(GO:0051260)[GSEA]


|  || Dataset | readcount.sample |
| Phenotype | sample.cls |
| Upregulated in class | TE1 |
| GeneSet | PROTEIN\_HOMOOLIGOMERIZATION(GO:0051260) |
| Enrichment Score (ES) | 0.5405558 |
| Normalized Enrichment Score (NES) | 1.962492 |
| Nominal p-value | 0.0 |
| FDR q-value | 0.20129898 |
| FWER p-Value | 0.494 |
Table: GSEA Results Summary

  

Fig 1: Enrichment plot: PROTEIN\_HOMOOLIGOMERIZATION(GO:0051260)
  
Profile of the Running ES Score & Positions of GeneSet Members on the Rank Ordered List

  

| PROBE | DESCRIPTION   (from dataset) | GENE SYMBOL | GENE\_TITLE | RANK IN GENE LIST | RANK METRIC SCORE | RUNNING ES | CORE ENRICHMENT || 1 | 103220400 | KCNG3 |  |  | 600 | 35.704 | 0.4275 | Yes |
| 2 | 103226786 | KCND2 |  |  | 867 | 4.515 | 0.4730 | Yes |
| 3 | 103220812 | KCNF1 |  |  | 1205 | 3.018 | 0.4963 | Yes |
| 4 | 103238468 | KCNA4 |  |  | 2073 | 2.035 | 0.4835 | Yes |
| 5 | 103247014 | LOC103247014 |  |  | 2371 | 1.907 | 0.4945 | Yes |
| 6 | 103234462 | KCTD15 |  |  | 2391 | 1.899 | 0.5178 | Yes |
| 7 | 103227813 | KCTD6 |  |  | 2579 | 1.828 | 0.5327 | Yes |
| 8 | 103244284 | KCNG1 |  |  | 3208 | 1.664 | 0.5258 | Yes |
| 9 | 103230091 | KCTD3 |  |  | 3731 | 1.569 | 0.5224 | Yes |
| 10 | 103222607 | KCTD1 |  |  | 3770 | 1.562 | 0.5406 | Yes |
| 11 | 103231190 | CRTC3 |  |  | 4317 | 1.483 | 0.5350 | No |
| 12 | 103248102 | KCTD14 |  |  | 5447 | 1.355 | 0.5018 | No |
| 13 | 103215505 | KCTD9 |  |  | 5740 | 1.327 | 0.5056 | No |
| 14 | 103214593 | KCTD12 |  |  | 6578 | 1.255 | 0.4841 | No |
| 15 | 103224214 | KCND3 |  |  | 8763 | 1.100 | 0.4005 | No |
| 16 | 103220750 | KCNS3 |  |  | 9876 | 1.030 | 0.3638 | No |
| 17 | 103244566 | KCNB1 |  |  | 10352 | 1.001 | 0.3553 | No |
| 18 | 103248106 | KCTD21 |  |  | 10672 | 0.979 | 0.3535 | No |
| 19 | 103237307 | KCNV1 |  |  | 11819 | 0.905 | 0.3138 | No |
| 20 | 103242612 | TNFAIP1 |  |  | 11840 | 0.904 | 0.3244 | No |
| 21 | 103214441 | TRIM13 |  |  | 11976 | 0.896 | 0.3298 | No |
| 22 | 103243061 | KCTD2 |  |  | 12310 | 0.875 | 0.3260 | No |
| 23 | 103239088 | KCTD10 |  |  | 12453 | 0.866 | 0.3307 | No |
| 24 | 103223939 | CRTC2 |  |  | 12794 | 0.845 | 0.3262 | No |
| 25 | 103223265 | KCTD17 |  |  | 12984 | 0.834 | 0.3284 | No |
| 26 | 103230721 | KCTD13 |  |  | 13838 | 0.779 | 0.3001 | No |
| 27 | 103231951 | KCND1 |  |  | 13953 | 0.771 | 0.3048 | No |
| 28 | 103235074 | KCNC3 |  |  | 14879 | 0.715 | 0.2726 | No |
| 29 | 103234193 | CRTC1 |  |  | 15023 | 0.706 | 0.2752 | No |
| 30 | 103231707 | KCTD5 |  |  | 15639 | 0.666 | 0.2561 | No |
| 31 | 103245465 | KCNS1 |  |  | 15944 | 0.644 | 0.2507 | No |
| 32 | 103234709 | SHKBP1 |  |  | 16366 | 0.616 | 0.2397 | No |
| 33 | novel.369 | - |  |  | 16754 | 0.587 | 0.2299 | No |
| 34 | 103246586 | KCTD7 |  |  | 17492 | 0.532 | 0.2037 | No |
| 35 | 103233285 | KCTD19 |  |  | 18081 | 0.482 | 0.1836 | No |
| 36 | 103244724 | KCTD16 |  |  | 18490 | 0.441 | 0.1709 | No |
| 37 | 103242292 | KCTD11 |  |  | 19836 | 0.298 | 0.1146 | No |
| 38 | 103224255 | KCNC4 |  |  | 21191 | 0.000 | 0.0540 | No |
| 39 | 103248466 | LOC103248466 |  |  | 21280 | 0.000 | 0.0501 | No |
| 40 | 103235018 | KCNA7 |  |  | 21491 | 0.000 | 0.0407 | No |
| 41 | 103246157 | KCTD8 |  |  | 21837 | 0.000 | 0.0253 | No |
Table: GSEA details
[plain text format]

  

Fig 2: PROTEIN\_HOMOOLIGOMERIZATION(GO:0051260)
  
Blue-Pink O' Gram in the Space of the Analyzed GeneSet

  

Fig 3: PROTEIN\_HOMOOLIGOMERIZATION(GO:0051260): Random ES distribution
  
Gene set null distribution of ES for
 **PROTEIN\_HOMOOLIGOMERIZATION(GO:0051260)**
